# Supplementary material for: Extended Shine-Dalgarno motifs govern translation initiation in Staphylococcus aureus
Source: Nat Commun. 2026 Feb 12;17:2678. doi: 10.1038/s41467-026-69079-8 (PMC13009471; doi:10.1038/s41467-026-69079-8)
Supplement: Supplementary file 1 — Supplementary Information [file 41467_2026_69079_MOESM1_ESM.pdf]

Supplementary information for:

Extended Shine-Dalgarno motifs govern translation initiation in *Staphylococcus aureus*
Kohl et al., (2026)

This document includes the following sections:

Supplementary figures S1-S8 with their respective figure legends.

Table S1. List of oligonucleotides used in this study, related to Methods.

Table S2. Cryo-EM data processing statistics, related to Methods.

Table S3. Resource table.

Note: The full list of sORFs identified in this study is provided as a separate document (**Supplementary Data 1**).

Figure S1.

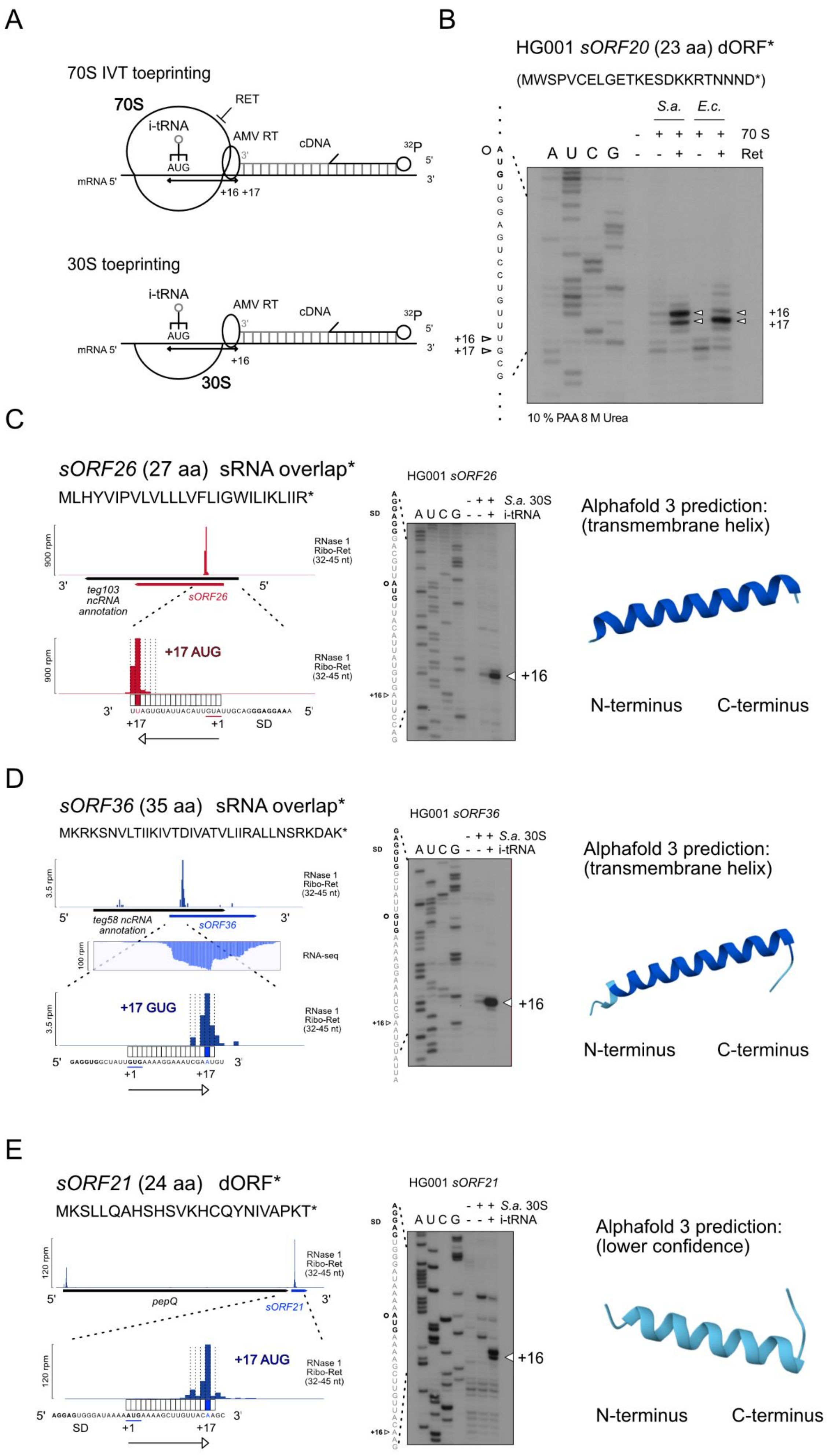
A
70S IVT toeprinting
70S
RET
i-tRNA
AMV RT
cDNA
AUG
mRNA 5'
+16 +17
30S toeprinting
30S
+16
B
HG001 sORF20 (23 aa) dORF*
(MWSPVCELGETKESDKKRTNNND*)
S.a.
E.c.
70 S
Ret
A U C G
+16
+17
10 % PAA 8 M Urea
C
sORF26 (27 aa) sRNA overlap*
MLHYVIPVLVLLLVFLIGWILIKLIIR*
RNase 1 Ribo-Ret (32-45 nt)
900 rpm
teg103 ncRNA annotation
sORF26
+17 AUG
SD
HG001 sORF26
S.a. 30S
i-tRNA
Alphafold 3 prediction:
(transmembrane helix)
N-terminus
C-terminus
D
sORF36 (35 aa) sRNA overlap*
MKRKSNVLTIIKIVTDIVATVLIIRALLNSRKDAK*
3.5 rpm
teg58 ncRNA annotation
sORF36
RNA-seq
100 rpm
+17 GUG
HG001 sORF36
Alphafold 3 prediction:
(transmembrane helix)
N-terminus
C-terminus
E
sORF21 (24 aa) dORF*
MKSLLQAHSHSVKHCQYNIVAPKT*
120 rpm
pepQ
sORF21
+17 AUG
HG001 sORF21
Alphafold 3 prediction:
(lower confidence)
N-terminus
C-terminus

Figure S1. Biochemical validation of initiation complex formation on novel sORF candidate sequences, related to Figure 1.

(A) Graphical illustration of toeprinting analysis for monitoring of translation initiation complex formation. Initiation complexes were formed on candidate sORF sequences either with purified *S. aureus* 70S ribosomes, trapped by addition of Retapamulin (Ret) during *in vitro* translation, or with 30S subunits. Primer extension inhibition analysis of radiolabeled oligonucleotides was used to assess the position and efficiency of initiation complex formation.

(B) 70S initiation complex toeprinting analysis of candidate *sORF20*, referenced in main figure 1E. Toeprints at positions +16 and +17 in respect to the predicted AUG start codon of *sORF20*, sequencing lanes (A, U, C, G), and the corresponding nucleotide sequence are indicated. Toeprints obtained with either *S. aureus* or *E. coli* purified 70S are shown.

(C, D, E) Ribo-Ret profiles of *S. aureus sORF26* (C), *sORF36* (D) and *sORF21* (E), displaying ribosomal density peaks and a zoomed focus on their respective initiation sites. Toeprinting analyses indicating 30S initiation complex formation corresponding to each of the Ribo-Ret footprints are shown on the right of each panel, next to a structural model of the designated small proteins as predicted by AlphaFold. The respective small protein sequence is indicated on top of each Ribo-Ret density profile. For each 30S toeprint, sequencing lanes and the corresponding nucleotide sequence are displayed.

Figure S2. STAR (*S. aureus* repeat) motifs recruit ribosomes for translation initiation at their conserved 5' sequences, designating sORFs of variable length, related to Figure 1.

(A) Exemplary Ribo-seq and Ribo-Ret profiles of *sORF1*, corresponding to a 5 aa short sORF, whose initiation site is one of several conserved STAR motif 5' sequences. The exact position of the Ribo-Ret footprint is shown on a zoomed view at the bottom, highlighting 3' end density at position +16 in respect to an AUG start codon.

(B) Sequence comparison of the RBS motifs of STAR sequences, displaying Ribo-Ret densities and designating variable sORFs, expressed in our experimental conditions. The selected start codons (blue) with appropriately spaced purine-rich tracts (bold) are highlighted in the alignments and a corresponding sequence motif is displayed below. The different small proteins designated by the five STAR sequences shown, are indicated below their respective sORF identifiers, in gray.

Figure S2.

A

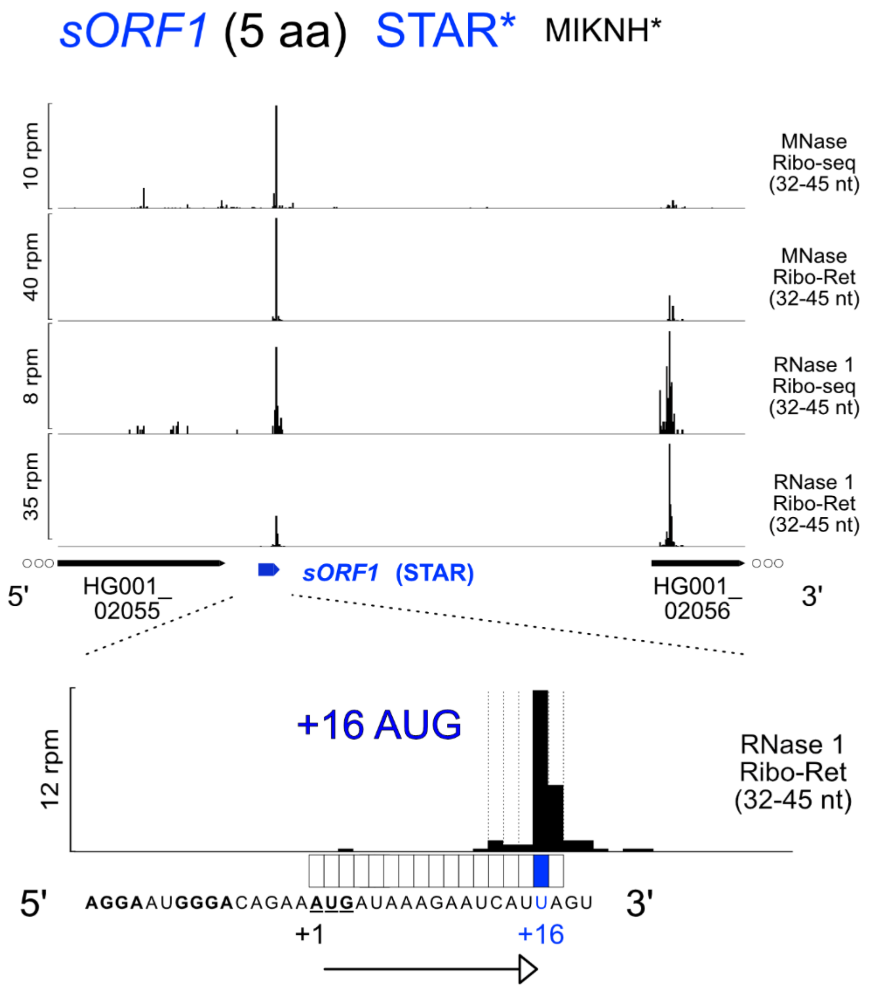

B

The start sequence of STAR (*S. aureus* repeat) motifs harbors a conserved purine rich tract and start codon, consistently recruiting ribosome density.

sORF1 UA**GGA**AU**GGGA**CAGAAAUGAUAAAGAAUCAUUAG
*MIKNH**

sORF2 **GGGGAG**U**GGGA**CAGAAAUGAUAAAGAGCCACUAA
*MIKNH**

sORF32 **GGGGAG**U**GGGA**CAGAAAUGAUAUUUUCGCAAAAU
*MIFSQNLFRRPTPTCIVCRNWESNFSLLGPRRQG**

sORF38 A**GGGAG**U**GGGA**CAUAAAUGAUAUUUUCGCAAAAU
*MIFSQNLFRRPTPTCIVCRNWESNFSLLGPIPNLHCL**

sORF39 **GGGGAG**U**GGGA**CAGAAAUGAUAUUUUCGCAAAAU
*MIFSQNLFRRPTPTCIVCRNWESNFSLLGPRPNSHCL**

RGGARUGGGACA - AA AUG (motif)

Figure S3.

Ribo-Ret expression data filtering for discovery of sORFs either missing in our HG001 annotation or annotated as hypothetical proteins

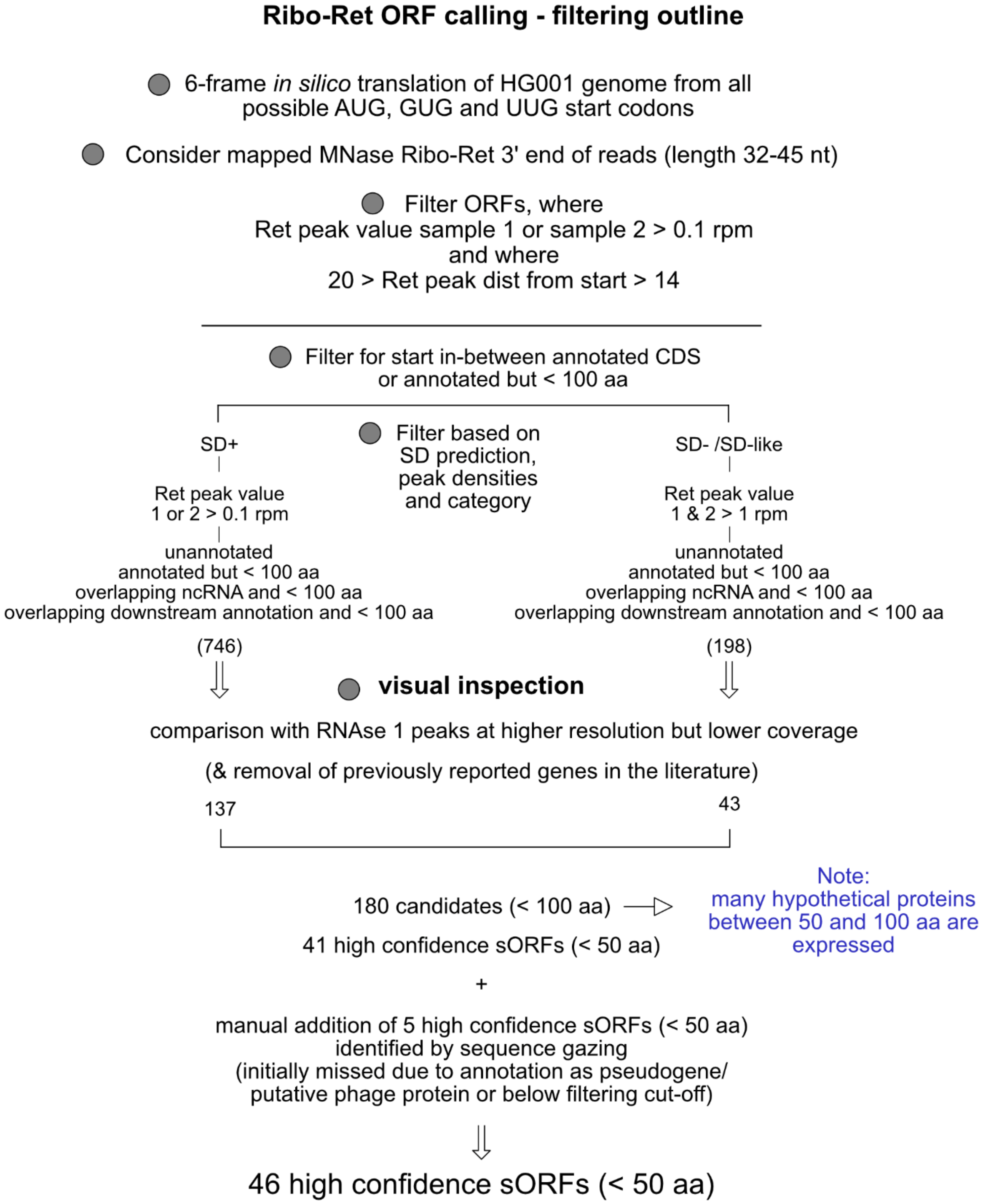

Figure S3: Ribo-Ret expression data filtering scheme for sORF discovery in *S. aureus* HG001, related to Methods and Figure 1.

Schematic flow chart representation of the analysis performed to identify sORFs by filtering of mapped Ribo-Ret expression data. Initially, all possible sORFs from AUG, GUG or UUG start codons were *in silico* translated into candidate sequences. Subsequently, they were either kept or discarded based on whether Ribo-Ret density peaks were adequately positioned in respect to the putative start sites and above indicated rpm expression thresholds. Key decisions during the analysis with this filtering approach are indicated by grey circles. Differential criteria considered for filtering, such as predicted SD motif presence are designated on top of each branch of the decision tree. The number of candidate- sORFs remaining after computational filtering and after visual inspection are indicated accordingly.

Figure S4: High-resolution Ribo-Ret facilitated improved genome annotation, resulting in minor and major start codon re-assignments, related to Figures 1 and 2.

(A) Exemplary Ribo-seq and Ribo-Ret profiles of *sepA*, with density peaks, whose positions designate alternative in-frame start codon selection. An appropriately spaced GUG start codon (blue), was re-annotated compared to the previously annotated AUG start codon (orange). This example is representative of several minor re-annotations as a result of improved resolution by RNase 1 Ribo-Ret.

(B) Exemplary Ribo-Ret profiles of *sarX*, with RPF density peaks far from the previously annotated putative initiation site, designating a major re-annotation. The start site of the previous annotation is indicated in orange, while the new annotation at an appropriately spaced UUG start codon with strong Ribo-Ret peak, is shown in blue. The SD sequence is shown in bold.

Figure S4.

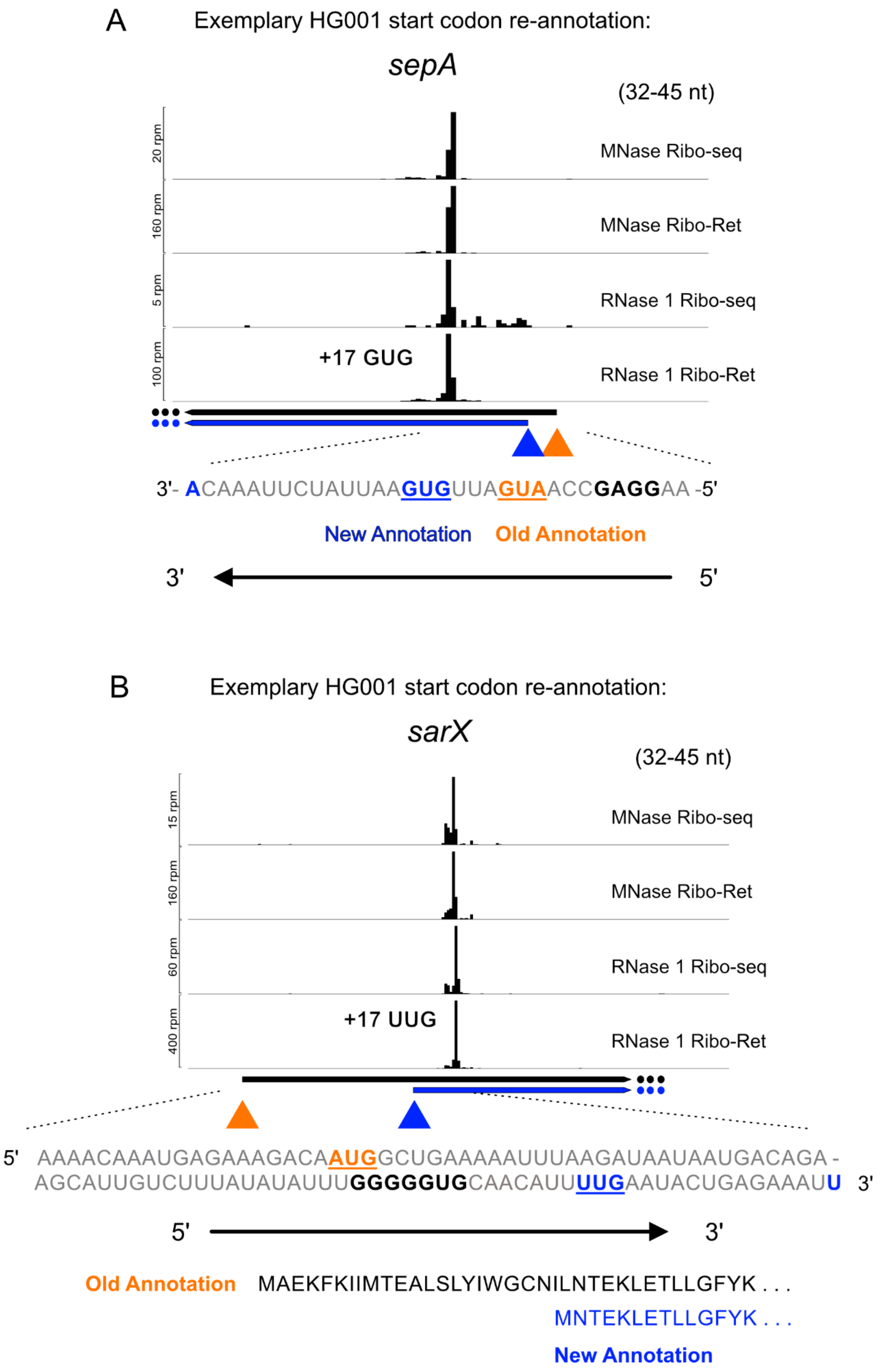
A
Exemplary HG001 start codon re-annotation:
sepA
(32-45 nt)
20 rpm
MNase Ribo-seq
160 rpm
MNase Ribo-Ret
5 rpm
RNase 1 Ribo-seq
100 rpm
+17 GUG
RNase 1 Ribo-Ret
3'- ACAAAUUCUAUUAAGUGUUAGUAACCGAGGAA -5'
New Annotation
Old Annotation
3'
5'
B
Exemplary HG001 start codon re-annotation:
sarX
(32-45 nt)
15 rpm
MNase Ribo-seq
160 rpm
MNase Ribo-Ret
60 rpm
RNase 1 Ribo-seq
400 rpm
+17 UUG
RNase 1 Ribo-Ret
5' AAAACAAAUGAGAAAGACAAUGGCUGAAAAAUUUAAGAUAAUAAUGACAGA -
AGCAUUGUCUUUAUAUAUUUGGGGGUGCAACAUUUUGAAUACUGAGAAAUU 3'
5'
3'
Old Annotation
MAEKFKIIMTEALSLYIWGCNILNTEKLETLLGFYK . . .
MNTEKLETLLGFYK . . .
New Annotation

Figure S5.

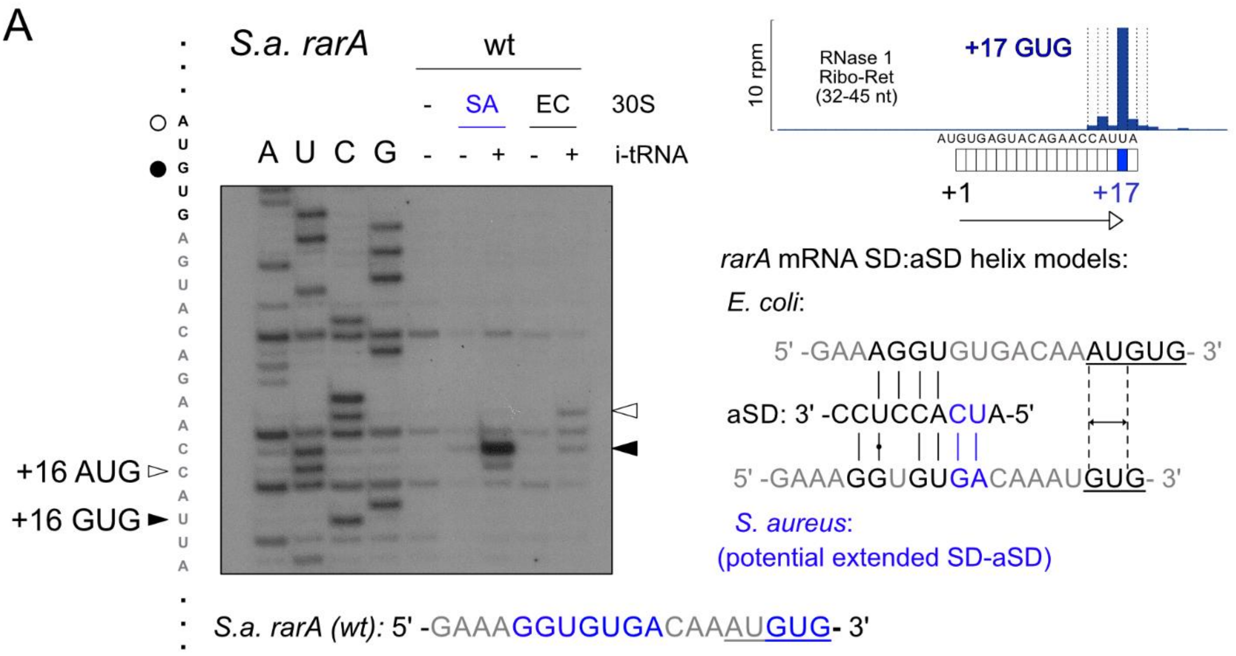
A
S.a. rarA
wt
- SA EC 30S
A U C G - - + - + i-tRNA
+16 AUG
+16 GUG
10 rpm
RNase 1
Ribo-Ret
(32-45 nt)
+17 GUG
AUGUGAGUACAGAACCAUUA
+1
+17
rarA mRNA SD:aSD helix models:
E. coli:
5' -GAAAGGUGUGACAAAUGUG- 3'
aSD: 3' -CCUCCACUA-5'
5' -GAAAGGUGUGACAAAUGUG- 3'
S. aureus:
(potential extended SD-aSD)
S.a. rarA (wt): 5' -GAAAGGUGUGACAAAUGUG- 3'

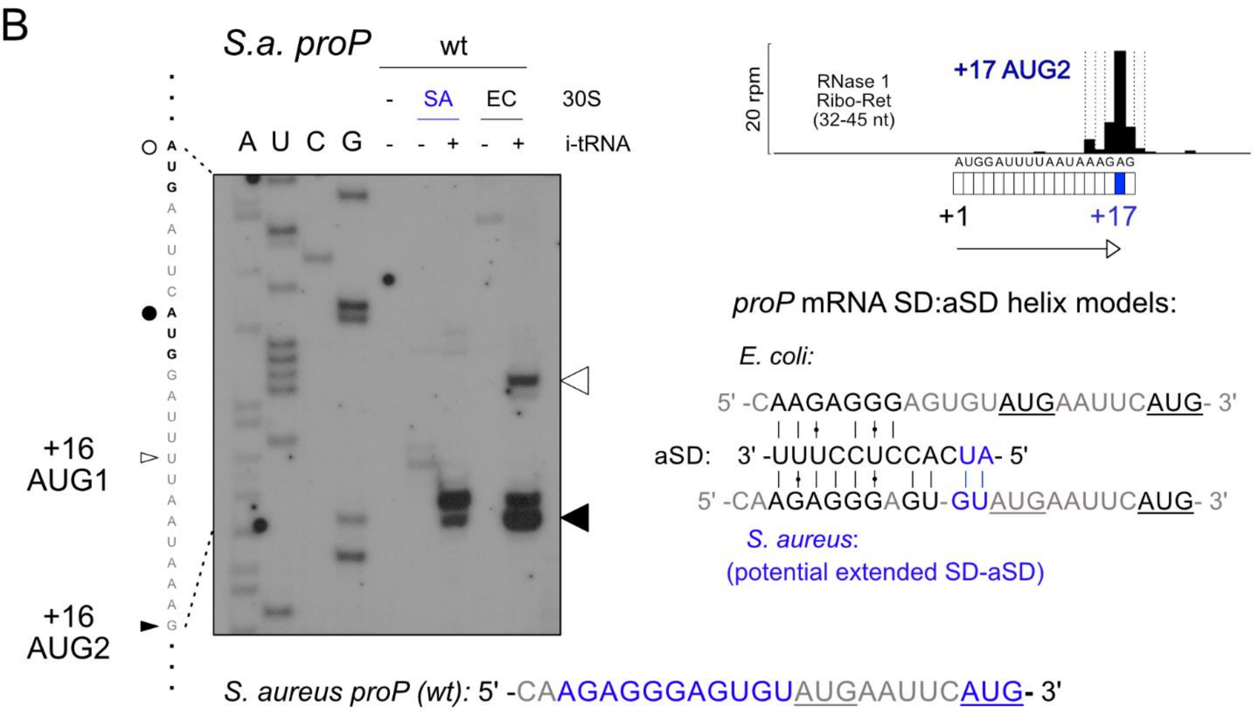
B
S.a. proP
wt
- SA EC 30S
A U C G - - + - + i-tRNA
+16
AUG1
+16
AUG2
20 rpm
RNase 1
Ribo-Ret
(32-45 nt)
+17 AUG2
AUGGAUUUUAAUAAAGAG
+1
+17
proP mRNA SD:aSD helix models:
E. coli:
5' -CAAGAGGGAGUGUAUGAAUUCAUG- 3'
aSD: 3' -UUUCCUCCACUA- 5'
5' -CAAGAGGGAGU-GUAUGAAUUCAUG- 3'
S. aureus:
(potential extended SD-aSD)
S. aureus proP (wt): 5' -CAAGAGGGAGUGUAUGAAUUCAUG- 3'

Figure S5: Alternative SD-aSD alignment during translation initiation through extended SD-aSD formation can direct differential start codon selection between *S. aureus* and *E. coli*, related to Figure 3.

(A, B) Comparative 30S toeprinting analysis of differential start codon selection by *S. aureus* and *E. coli* ribosomes on the natural *S. aureus* mRNA sequences *rarA* (A) and *proP* (B). For both mRNA's SD motifs, extended SD-aSD interactions are predicted to form. The predicted model for *rarA* is displayed on the right of the toeprint panel, below its RNase 1 Ribo-Ret density profile (A). An extended interaction may differently align the SD and aSD sequences on this particular mRNA. In panel B, the model for the SD-aSD interaction on *proP*, shown below its Ribo-Ret density profile, indicates similar alignments between *S. aureus* and *E. coli*. However, the possible extended interactions may preclude initiation at the first of two potential AUG start codons. For both panels, toeprint bands at positions +16 (arrows) in respect to either potential start codon, sequencing lanes and the corresponding nucleotide sequences are indicated. For both *rarA* and *proP*, the full RBS sequence is displayed below the toeprint gel, highlighting the extended SD motif and the in-frame start codon in *S. aureus* in blue.

Figure S6.

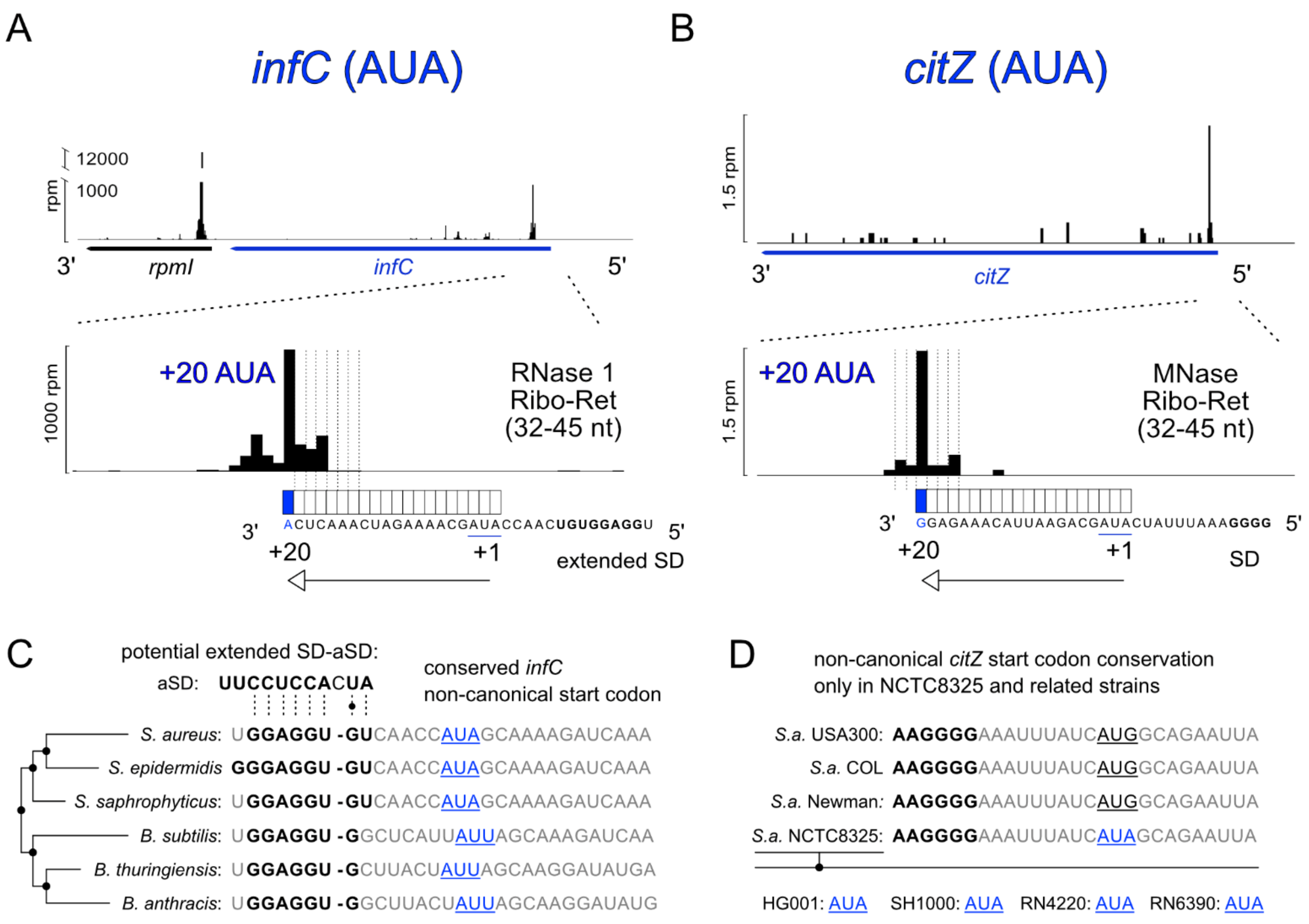

Figure S6: *infC* and *citZ* utilize non-canonical AUA start codons in *S. aureus* HG001 strain, related to Figure 4.

(A, B) Non-canonical start codon usage by the *infC* (A) and *citZ* (B) mRNAs is shown, evidenced by their respective Ribo-Ret expression profiles. The Ribo-Ret data type used for visualization is indicated, e.g., indicating the use of MNase Ribo-Ret data of read lengths 32-45 nt for *citZ* (B) due to low expression values and sequencing coverage of the locus.

(C, D) RBS sequence conservation analysis of *infC* (C) and *citZ* (D), as performed by comparison of curated genome information extracted from the SEED database. Potential SD-aSD motif interactions are shown in bold, including putative extended interactions in the case of *infC* (C), while conserved non-canonical start codons are highlighted in blue.

Figure S7.

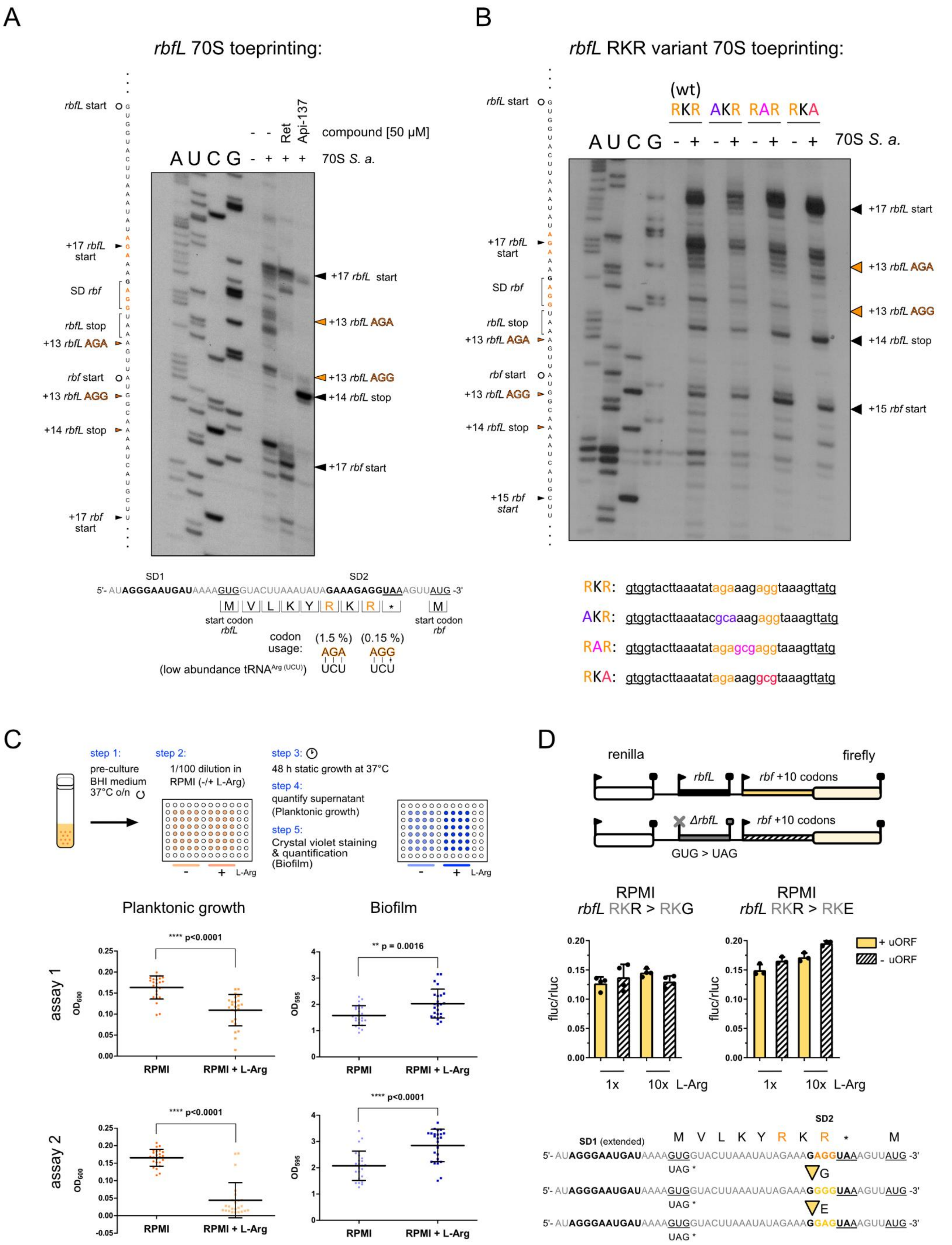
A
rbfL 70S toeprinting:
compound [50 µM]
70S S. a.
A U C G
Ret
Api-137
+17 rbfL start
+13 rbfL AGA
+13 rbfL AGG
+14 rbfL stop
+17 rbf start
SD rbf
rbfL stop
rbf start
SD1
SD2
5'- AUAGGGAAUGAUAAAAGUGGUACUUAAAUAUAGAAAGAGGUAAAGUUAUG -3'
M V L K Y R K R * M
start codon rbfL
start codon rbf
codon usage: (1.5 %) AGA (0.15 %) AGG
(low abundance tRNA Arg (UCU)) UCU UCU
B
rbfL RKR variant 70S toeprinting:
(wt) RKR AKR RAR RKA
70S S. a.
+17 rbfL start
+13 rbfL AGA
+13 rbfL AGG
+14 rbfL stop
+15 rbf start
RKR: gtggtacttaaatatagaaagaggtaaagttatg
AKR: gtggtacttaaatacgcaaagaggtaaagttatg
RAR: gtggtacttaaatatagagcgaggtaaagttatg
RKA: gtggtacttaaatatagaaaggcgtaaagttatg
C
step 1: pre-culture BHI medium 37°C o/n
step 2: 1/100 dilution in RPMI (-/+ L-Arg)
step 3: 48 h static growth at 37°C
step 4: quantify supernatant (Planktonic growth)
step 5: Crystal violet staining & quantification (Biofilm)
- + L-Arg
Planktonic growth
Biofilm
assay 1
assay 2
**** p<0.0001
** p = 0.0016
OD600
OD595
RPMI
RPMI + L-Arg
D
renilla
firefly
rbfL
rbf +10 codons
ΔrbfL
GUG > UAG
RPMI rbfL RKR > RKG
RPMI rbfL RKR > RKE
fluc/rluc
+ uORF
- uORF
1x 10x L-Arg
SD1 (extended)
SD2
M V L K Y R K R * M
5'- AUAGGGAAUGAUAAAAGUGGUACUUAAAUAUAGAAAGAGGUAAAGUUAUG -3'
UAG *
G
5'- AUAGGGAAUGAUAAAAGUGGUACUUAAAUAUAGAAAGGGGUAAAGUUAUG -3'
E
5'- AUAGGGAAUGAUAAAAGUGGUACUUAAAUAUAGAAAGGAGUAAAGUUAUG -3'

Figure S7: *rbfL* translation pauses at rare Arg codons and biofilm formation is promoted by exogenous L-Arg, related to Figure 6.

(A, B) Toeprinting analysis of the native (A) and mutant (B) *rbfL* sequence context during *in vitro* translation by *S. aureus* 70S ribosomes. The absence or presence of translation inhibitors Retapamulin (Ret) and Apidaecin-137 (Api-137) as well as sequencing lanes (A, U, C, G) and toeprint positions are indicated accordingly. Orange triangles mark pause signatures at +13 relative to the rare Arg codons AGA and AGG at the expected distance for decoding in the A-site. The nucleotide contexts for the wild type and alanine-scan variants (RKR→AKR/RAR/RKA) are shown below the gel in panel B.

(C) The results from two independent assays quantifying the effect of exogenous L-Arg supplementation to biofilm formation in poor RPMI media are shown. The assay's workflow is depicted schematically, indicating steps from pre-culture conditions to static growth for 48 h and quantification of the supernatant (planktonic growth) prior to the quantification of biofilm through Crystal Violet staining. For each assay and condition, 24 technical replicates were performed and the mean and standard deviation are shown, overlaying the individual datapoints. Two-tailed unpaired t-test, $***p < 0.001$. Calculated p-values for significant differences in the comparison of planktonic growth with and without L-Arg supplementation in assays one and two were < 0.0001 in both cases, while p-values for biofilm formation with and without L-Arg supplementation in assays one and two were 0.0016 and < 0.0001, respectively.

(D) *In vivo* dual luciferase assays analyzing the contribution of the *rbfL* RKR (arginine-lysine-arginine) motif to *rbf* regulation in RPMI ± L-Arg. The rare AGG codon in *rbfL* was replaced with GGG (RKG; Gly) or GAG (RKE; Glu) while preserving a strong SD for *rbf* (schematics below). Bars show normalized Firefly/Renilla expression for reporters with (+uORF) or without (−uORF) the *rbfL* start codon (GUG→UAG). The mean ± standard deviation is shown from four biological replicates in case of RKR>RKG mutant analysis and from three biological replicates in case of RKR>RKE mutant analysis. Both substitutions abolish *rbfL*-dependent repression and render *rbf* expression insensitive to L-Arg.

Figure S8.

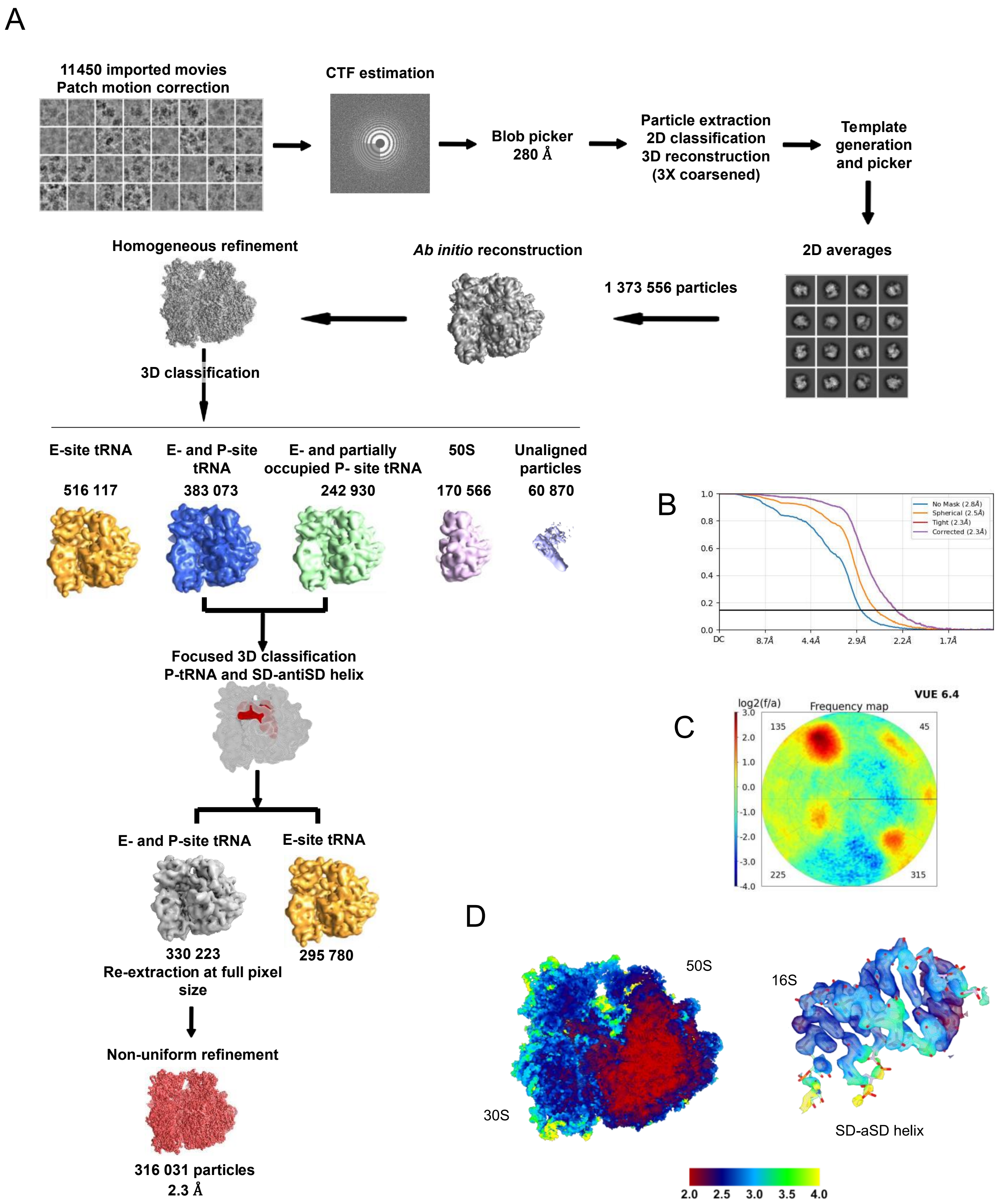
A
11450 imported movies
Patch motion correction
CTF estimation
Blob picker
280 Å
Particle extraction
2D classification
3D reconstruction
(3X coarsened)
Template
generation
and picker
2D averages
1 373 556 particles
Ab initio reconstruction
Homogeneous refinement
3D classification
E-site tRNA
516 117
E- and P-site
tRNA
383 073
E- and partially
occupied P- site tRNA
242 930
50S
170 566
Unaligned
particles
60 870
Focused 3D classification
P-tRNA and SD-antiSD helix
E- and P-site tRNA
E-site tRNA
330 223
Re-extraction at full pixel
size
295 780
Non-uniform refinement
316 031 particles
2.3 Å
B
No Mask (2.8Å)
Spherical (2.5Å)
Tight (2.3Å)
Corrected (2.3Å)
DC
8.7Å
4.4Å
2.9Å
2.2Å
1.7Å
C
VUE 6.4
log2(f/a)
Frequency map
135
45
225
315
D
50S
30S
16S
SD-aSD helix
2.0
2.5
3.0
3.5
4.0

Figure S8: Single particle cryo-EM data processing of the *S. aureus* 70S initiation complex, related to Methods and Figure 3.

(A) Processing workflow. Representative micrographs, CTF estimates, and particle selection are shown, followed by 2D classification, *ab initio* 3D reconstruction and 3D classification into the major classes. Particles from the non-rotated 70S classes (E/P and E/partially occupied P) were subjected to focused 3D classification on the P-site tRNA and SD-aSD helix, then refined to yield the final map.

(B) FSC curves for the final reconstruction (unmasked, masked, and corrected), with the 0.143 criterion indicated (black line). The final masked resolution is ~2.3 Å.

(C) Angular view distribution of particles in the final map, rendered with VUE software, illustrating the sampling of orientations of views.

(D) Local resolution estimated with Cryosparc 4.7.1 software. Left: 70S initiation complex. Right: Extended Shine-Dalgarno- anti Shine-Dalgarno helix.

Table S1. List of oligonucleotides used in this study, related to Methods.

Name	Sequence 5'-3'	Purpose
oMK53	TAATACGACTCACTATAggatagtttaattaaactgactaca	sORF5 T7 fwd
oMK54	gctactcaaatacaacatgatcg	sORF5 T7 rev
oMK55	caaaataagactatgccatcttg	sORF5 toeprinting
oMK65	TAATACGACTCACTATAgtggtaaatgtttgataaaatagtat	sORF7 T7 fwd
oMK66	tattaatgtttcgttatccgcc	sORF7 T7 rev
oMK67	attgttccattgtaatctccct	sORF7 toeprinting
oMK128	TAATACGACTCACTATAgcatttttgtaataacttctcaaag	sORF9 T7 fwd
oMK129	gacttacattaacagcactcg	sORF9 T7 rev
oMK130	cataaagcgttaatcttcccttt	sORF9 toeprinting
oMK56	TAATACGACTCACTATAgacataaaaatgtggattctagg	sORF15 T7 fwd
oMK57	ggatttttgagccatagcat	sORF15 T7 rev
oMK58	gactcttaactgttatttacatttac	sORF15 toeprinting
oMK59	TAATACGACTCACTATAgacatcaagatgtggattctagg	sORF16 T7 fwd
oMK60	gcttttgatttgcccatttac	sORF16 T7 rev
oMK61	gactcttaactattacttacacttac	sORF16 toeprinting
oMK11	TAATACGACTCACTATAGaataatttgaaatgattagcgtatac	sORF18 T7 fwd
oMK12	gttgataattagcattttccctag	sORF18 T7 rev
oMK15	gataaacaaattgataattattcgag	sORF18 toeprinting
oMK110	TAATACGACTCACTATAgaaataaactagaaaatatagtatcattatgg	sORF20 T7 fwd
oMK111	cacaaaaaaataaccacactcc	sORF20 T7 rev
oMK112	gtttaaaaattagtcgttattgttagt	sORF20 toeprinting
oMK98	TAATACGACTCACTATAggcctaggattacaagaaca	sORF21 T7 fwd
oMK99	gttacatattgcatttagacttgg	sORF21 T7 rev
oMK100	ggataaaaatttatgtcttaggcg	sORF21 toeprinting
oMK101	TAATACGACTCACTATAgaagtggtatcataccgatatg	sORF22 T7 fwd
oMK102	gtgaagctaaaataactacaaatga	sORF22 T7 rev
oMK103	gtatataaatttcacttcccatgg	sORF22 toeprinting
oMK263	TAATACGACTCACTATAgccatattcatattacttctcatcg	sORF25 T7 fwd
oMK264	catgatttctttcacttttagaaaac	sORF25 T7 rev
oMK265	ggcgaaatttttaattttttagaatatc	sORF25 toeprinting

oMK95	TAATACGACTCACTATAgaagttaaaggagggacgttat	sORF26 T7 fwd
oMK96	cacatccgaatattacaaacac	sORF26 T7 rev
oMK97	gcggataattaactttattaaaatcc	sORF26 toeprinting
oMK1	TAATACGACTCACTATAgtaagtttaagatatgttgtatagg	sORF27 T7 fwd
oMK2	gaattctatcagccgcattatc	sORF27 T7 rev
oMK5	gagctaaagcgattgc	sORF27 toeprinting
oMK86	TAATACGACTCACTATAgttgaatggtgagcttattggg	sORF36 T7 fwd
oMK87	cgactcgattacttggtattaaaa	sORF36 T7 rev
oMK88	aagcacgtatgattaacacg	sORF36 toeprinting
oMK134	TAATACGACTCACTATAggcggatggtttagtaaatg	sORF45 T7 fwd
oMK135	cttccacgccatataatttca	sORF45 T7 rev
oMK136	cttacgagtaaatgttccatataaaa	sORF45 toeprinting
oMK526	TAATACGACTCACTATAgatttcaggaggaatgaaatgtgaggaaattttc	aur wt T7 fwd
oMK527	TAATACGACTCACTATAgatttcaggaggaatgatgtgaggaaattttc	aur spacer -2 T7 fwd
oMK528	TAATACGACTCACTATAgatttcaggaggaatgaaAatgtgaggaaattttc	aur spacer +1 T7 fwd
oMK529	TAATACGACTCACTATAgatttcaggaggaatgaaAAatgtgaggaaattttc	aur spacer +2 T7 fwd
oMK530	TAATACGACTCACTATAgatttcaggaggaCCgaaatgtgaggaaattttc	aur delta extended SD T7 fwd AU>CC
oMK554	TAATACGACTCACTATAgatttcaggaggaCtgaaatgtgaggaaattttc	aur delta extended SD T7 fwd AU>CU
oMK555	TAATACGACTCACTATAgatttcaggaggaaCgaaatgtgaggaaattttc	aur delta extended SD T7 fwd AU>AC
oMK556	TAATACGACTCACTATAgatttcaggaggaGtgaaatgtgaggaaattttc	aur delta extended SD T7 fwd AU>GU
oMK78	catcagtaacagcgtaatcttg	aur T7 rev
oMK79	cgctaatgctgctggtg	aur toeprinting
oMK122	TAATACGACTCACTATAgcgtaacgcgaaaagtc	rlmB wt T7 fwd
oMK123	gaacaatgattttttgatcttttgc	rlmB T7 rev
oMK124	caatatcttatttatcggatgccc	rlmB toeprinting
oMK535	TAATACGACTCACTATAGatacaggggggtattaatttgaaaaagaaaaac	spa wt T7 fwd
oMK537	TAATACGACTCACTATAGatacaggggggtattaatGtgaaaaagaaaaac	spa mutant AUGUG T7 fwd

Romby-lab oligo 1287	TTTGCAGCAGGTGTTACGCC	spa T7 rev and toeprinting
oMK154	TAATACGACTCACTATAgaaaggtgtgacaaatgtgag	rarA T7 fwd
oMK155	gcttcttcaacaacaagttg	rarA T7 rev
oMK162	ggtccaactaaatgttgttgg	rarA toeprinting
oMK74	TAATACGACTCACTATAgatacaagagggagtgtatgaatt	proP T7 fwd
oMK75	ggctaatgctgcgaaag	proP T7 rev
oMK76	gcattaccgataccggt	proP toeprinting
oMK331	aatagcgaagaggcccgc	Luciferase cassette transfer prab12 backbone fwd
oMK332	acgccagctggcgaaagg	Luciferase cassette transfer prab12 backbone rev
oMK333	cccctttcgccagctggcgtagcttactatgccattattaataacttag	Luciferase cassette fwd
oMK334	gtgcgggcctcttcgctattaaaaaagcaacgcactttaaataag	Luciferase cassette rev
oMK180	GAAGACGCCAAAAACATAAAG	prabm30-33 vector backbone fwd
oMK419	CTTGATGTCAGATCTTTATTGTTC	prabm30-33 vector backbone rev
oMK420	aataaagatctgacatcaagCATTTACTTTTCTGAAAAATTAAATTAATATTTTATTTATAAG	prabm30&31 aur insert fwd
oMK421	tttatgtttttggcgtcttcCACATTTCATTCCTCCTGAAATC	prabm30 aur insert rev
oMK422	tttatgtttttggcgtcttcGCACATTTCATTCCTCCTG	prabm31 aur insert rev
oMK423	aataaagatctgacatcaagGCGTAACGCGAAAAGTCATAC	prabm32&33 rlmB insert fwd
oMK424	tttatgtttttggcgtcttcCACATCGTCACTACCTTTCG	prabm 32 rlmB insert rev
oMK426	tttatgtttttggcgtcttcCCACATCGTCACTACCTTTC	prabm 33 rlmB insert rev
oMK479	gaagacgccaaaaacataaag	prabm 39, 40, 42, 68, 69, 70, 71 vector backbone fwd
oMK483	cttgatgtcagatctttattgttc	prabm 39, 40, 42, 68, 69, 70, 71 vector backbone rev
oMK484	aataaagatctgacatcaagatgtcaaatttaaaacagtaacacttatttac	prabm 39, 40, 42 cvfC1 insert fwd
oMK485	tttatgtttttggcgtcttcctctgaaaaagttaaaatacgcg	prabm39 cvfC1 insert rev

oMK486	tttatgtttttggcgtcttcctctgaaaaagttaacatacgc	prabm40 cvfC1 mut insert rev
oMK488	tttatgtttttggcgtcttcctctgaaaaagtttaaatacgcg	prabm42 cvfC1 stop insert rev
oMK567	tttatgtttttggcgtcttcttgactgagttctataggtcttcac	prabm68 ccpN wt insert fwd
oMK568	tttatgtttttggcgtcttcttgactgagttcCataggtcttcac	prabm69 ccpN AUG insert fwd
oMK566	aataaagatctgacatcaagcaagtaaaattaatgtgtttgtgac	prabm68&69 ccpN insert rev
oMK570	tttatgtttttggcgtcttcttgtaattctgctatgataaatttcc	prabm70 citZ wt insert fwd
oMK571	tttatgtttttggcgtcttcttgtaattctgcCatgataaatttcc	prabm71 citZ AUG insert fwd
oMK569	aataaagatctgacatcaagcacaaacgaaatgcgtttag	prabm70&71 citZ insert rev
oMK176	agtattGAAGACGCCAAAAACATAAAG	Q5 SDM fwd oligo for prabm152 gehA fluc fusion
oMK177	atacttATTTTGACTTTTCATCATTGTC	Q5 SDM rev oligo for prabm152 gehA fluc fusion
oMK180	GAAGACGCCAAAAACATAAAG	Q5 SDM fwd oligo for fluc fusion changes
oMK181	GATAAACAAATTGATAATTATTCGAG	Q5 SDM rev oligo for prabm154 gehA uORF (sORF18) fusion (with oMK180)
oMK193	AGATCTTTATTGTTCATTTTTGAG	prabm1515, prabm1521, prabm1524 vector backbone rev oligo (with oMK180)
oMK194	aaaatgaacaataaagatctGGATAGTTTAATTAAACTGACTAC	prabm1515 rbf insert fwd
oMK195	tttatgtttttggcgtcttcAGTAAGTATATGCAAGCATG	prabm1515 rbf insert rev
oMK196	AATGATAAAAtaGGTACTTAAATATAGAAAGAGG	prabm1516 q5 SDM oligo fwd
oMK197	CCCTATAATATTTAACTTATTTAATTAAAC	prabm1516 q5 SDM oligo rev

oMK198	CCTCTTTCTATATTTAAGTACC	prabm1517 q5 SDM oligo rev (with oMK180)
oMK199	agatctttattgttcatttttgagaac	prabm1518 vector backbone rev oligo (with oMK180)
oMK200	aaaatgaacaataaagatctgttaagaattataaaaatgtaaagtatatactttg	prabm1518 HG001_02379 insert fwd
oMK201	tttatgtttttggcgtcttccataaaacctacttgaggtaattg	prabm1518 HG001_02379 insert rev
oMK204	ATTATTGTTACCGTTATAATTTAC	prabm1520 q5 SDM oligo rev (with oMK180)
oMK205	aaaatgaacaataaagatctgcattttttgtaataacttctcaaag	prabm1521 pbp4 insert fwd
oMK206	tttatgtttttggcgtcttcaatgatgataatagatattaaatttttcataaag	prabm1521 pbp4 insert rev
oMK209	AAAGCAATGGTTATTCCTAC	prabm1523 q5 SDM oligo rev (with oMK180)
oMK210	aaaatgaacaataaagatctgacatcaagatgtggattc	prabm1524 HG001_01353 insert fwd
oMK211	tttatgtttttggcgtcttcgatgtatcttgttattacttttgc	prabm1524 HG001_01353 insert rev
oMK214	CTTACACTTACTAAACCAG	prabm1526 q5 SDM oligo rev (with oMK180)
oMK489	acagatctgagctcgaattcgtacttaactgtattttaaagagaaaagaaatac	prmp39, 40, 41 tRNA Arg(UCU) insert fwd oligo
oMK490	cttgggaggggctcacgaccgtgagttaaataattaaaaaaccacg	prmp39, 40, 41 tRNA Arg(UCU) insert rev oligo
oMK491	ggtcgtgagcccctcccaag	prmp39, 40, 41 vector backbone fwd oligo
oMK492	gaattcgagctcagatctgttaacgg	prmp39, 40, 41 vector backbone rev oligo
oMK497	gtacttaaatatagaaagGGGtaaagttatggcaaaatcatgc	prmp42&44 quickchange fwd
oMK498	gcatgattttgccataactttaCCCctttctatatttaagtac	prmp42&44 quickchange rev

oMK687	gtacttaaatatagaaagGGGgaagacgccaaaaacataaag	prabm91 quickchange fwd
oMK688	ctttatgtttttggcgtcttcCCCctttctatatttaagtac	prabm91 quickchange rev
oMK702	gtacttaaatatagaaagGAGtaaagttatggcaaaatcatgc	prabm98&99 quickchange fwd
oMK703	gcatgattttgccataactttaCTCctttctatatttaagtac	prabm98&99 quickchange fwd
oMK706	ggaatgataaaagtggtacttaaatatCGTaagaggtaaagttatggc	prabm101 quickchange fwd
oMK707	gccataactttacctcttACGatatttaagtaccacttttatcattcc	prabm101 quickchange rev
oMK708	ggaatgataaaaTAggtacttaaatatCGTaagaggtaaagttatggc	prabm102 quickchange fwd
oMK709	gccataactttacctcttACGatatttaagtaccTAttttatcattcc	prabm102 quickchange rev
oMK447	gggaatgataaaagtggtacttaaaTACGCAaagaggtaaagttatggc	prmp35 quickchange fwd
oMK448	gccataactttacctcttTGCGTAtttaagtaccacttttatcattccc	prmp35 quickchange rev
oMK449	gataaaagtggtacttaaatatagaGCGaggtaaagttatggcaaaatc	prmp36 quickchange fwd
oMK450	gattttgccataactttacctCGCtctatatttaagtaccacttttatc	prmp36 quickchange rev
oMK451	gataaaagtggtacttaaatatagaaagGCGtaaagttatggcaaaatcatg	prmp37 quickchange fwd
oMK452	catgattttgccataactttaCGCctttctatatttaagtaccacttttatc	prmp37 quickchange rev
oMK271	TAATACGACTCACTATAgatgcacctgatgaaatggg	PCR fwd oligo to amplify *rbfL* sequence variants from prab plasmids together with oMK272 for T7 IVT
oMK272	gcaactccgataaataacgc	PCR rev oligo to amplify *rbfL* sequence variants from prab plasmids together with oMK271 for T7 IVT

oMK440	cagcggatagaatggcg	toeprint oligo for *rbfL* sequence variants amplified from prab plasmids carrying Fluc reporter fusions

Table S2. Cryo-EM data processing statistics, related to Methods.

**Data collection processing**	
Magnification	165 000
Acceleration Voltage (kV)	300
Electron fluence (eÅ-^{2})	30
Defocus range (µm)	-0.8 to -2.8
Pixel size	0.729
Symmetry	C1
Initial particle images (no.)	1 931 605
Final particle images (no.)	316 031
Map resolution (Å)	2.3
Fourier Shell correlation threshold	0.143
**Refinement**	
Initial atomic model used (PDB code)	6YEF
Map/model resolution (0.5 FSC)	2.2
d_{99} resolution estimation	2.6
Map sharpening B factor (Å^{2})	36.9
Model composition	
Non-hydrogen atoms	142 927
Protein residues	5446
Nucleotides	4668
B factors (Å^{2})	
Protein	55
RNA	61
Root mean square deviations	
Bond lengths (Å)	0.005
Bond angles (Å)	0.594
Validation	
Molprobity score	1.70
Clashscore	5
Rotamer outliers	1.5
Ramachandran plot	
Favored (%)	96.67
Allowed (%)	3.25
Disallowed (%)	0.07

Table S3. Resource table.

REAGENT or RESOURCE	SOURCE	IDENTIFIER
Bacterial and virus strains		
S. aureus HG001	This study	N/A
S. aureus RN4220	This study	N/A
NEB 5-alpha *E. coli*	New England Biolabs	#C2987
Chemicals, peptides, and recombinant proteins		
Retapamulin	Euromedex	#TO-R027-25MG
RNase-free DNase 1	Sigma Aldrich	#04716728001
SuperaseIn RNase Inhibitor	Thermo Fisher	#AM2694
Rec RNase Inhibitor RNasin	Promega	#N251B
[gamma-P32] ATP	Hartmann Analytic	#SRP-501
GMPPNP	Sigma Aldrich	#G0635-5MG
MNase (Micrococcal nuclease S7)	Sigma Aldrich	#10107921001
RNase 1	New England Biolabs	#M0243S
RPMI media (1640 modified R7509)	Sigma Aldrich	#R7509
BHI media	Becton Dickinson	#237500
L-Amino acids (L-Arg used in this study)	Sigma Aldrich	#LAA21-1KT
T4 Polynucleotide Kinase	Thermo Fisher	#EK0032
Antarctic Phosphatase (AnP)	New England Biolabs	#M0289S
Lysing Matrix B	MP Biomedicals	#116911500
Triton X-100	Sigma Aldrich	#X100-100ML
NP-40 (Substitute)	Sigma Aldrich	#74385-1L
RNAPro Solution	MP Biomedicals	#116055050
Phusion High Fidelity DNA Polymerase	Life Technologies (Thermo Fisher Scientific)	#F-530XL
Ampicillin	Sigma Aldrich	#A9518-5G
Chloramphenicol	Sigma Aldrich	#C0378
Acidic PCI (phenol/chloroform/isoamyl alcohol)	Roth	#X985.3
PCI (phenol/chloroform/isoamyl alcohol)	Roth	#A156.1
Spermidine	Sigma Aldrich	#S0266
ATP	Sigma Aldrich	#A2383
GTP	Bio Basic Canada	#GD0250T
CTP	Sigma Aldrich	#C9274
UTP	Sigma Aldrich	#U6625-1G
DTT	Sigma Aldrich	#DO632
EDTA	Boehringer Ingelheim	#808261

Lysostaphine	Sigma Aldrich	#L9043-5MG
AMV RT	Promega	#M510F
PBS	Gibco (Thermo Fisher Scientific)	#14190-094
Dpn1	Thermo Fisher	#ER1702
Sucrose	Sigma Aldrich	#1076871000
Protease Inhibitor X100	Thermo Fisher	#78438
Critical commercial assays		
PURExpress Δ Ribosome Kit	New England Biolabs	#E3313S
Nucleospin Gel and PCR clean up	Macherey Nagel	#740609.250
Nucleospin Plasmid	Macherey Nagel	#740588.250
Dual-Glo® Luciferase Assay System	Promega	#E2920
NEBNext Small RNA Library Prep Set for Illumina	New England Biolabs	#E7560S
NEBNext Ultra II Directional RNA Library Prep Kit	New England Biolabs	#E7760
Staphylococcus aureus riboPOOL rRNA depletion kit	siTools	#dp-K024-21
NEBuilder HiFi DNA Assembly Cloning Kit	New England Biolabs	#E5520S
NEB Q5® Site-Directed Mutagenesis Kit	New England Biolabs	#E0554S
Deposited data		
Raw and processed Ribo-seq and RNA-seq data	This study	GEO: GSE299221 and GSE299222
Cryo-EM map	This study	EMDB: EMD-55526
S. aureus 70S initiation complex coordinates	This study	PDB: 9T4R
Oligonucleotides		
See table S1	This study	N/A
Recombinant DNA		
pCN43	Charpentier et al., (1)	N/A
prab11	Helle et al., (2)	N/A
prabm30 (*aur* GUG in-frame)	This study	N/A
prabm31 (*aur* AUG in-frame)	This study	N/A
prabm32 (*rlmB* GUG in-frame)	This study	N/A
prabm33 (*rlmB* AUG in-frame)	This study	N/A
prabm39 (*cvfC1* AUU)	This study	N/A
prabm40 (*cvfC1* AUG)	This study	N/A
prabm42 (*cvfC1* AUU-UAA stop)	This study	N/A
prabm68 (*ccpN* AUA)	This study	N/A
prabm69 (*ccpN* AUG)	This study	N/A
prabm70 (*citZ* AUA)	This study	N/A
prabm71 (*citZ* AUG)	This study	N/A

pmpk1 (dual luciferase plasmid with *gehA* initiation context for firefly luciferase)	Purchased from IGBMC molecular biology platform (Paola Rossolillo)	N/A
prabm154 (*sORF18*, *gehA* uORF fusion)	This study	N/A
prabm1518 (HG001_02379 fusion)	This study	N/A
prabm1520 (*sORF7*, HG001_02379 uORF fusion)	This study	N/A
prabm1524 (HG001_01353 fusion)	This study	N/A
prabm1526 (*sORF16*, HG001_01353 uORF fusion)	This study	N/A
prabm1521 (*pbp4* fusion)	This study	N/A
prabm1523 (*sORF9*, *pbp4* uORF fusion)	This study	N/A
prabm1517 (*sORF5*, *rbfL* fusion)	This study	N/A
prabm1515 (*rbf* fusion)	This study	N/A
prabm1516 (*rbf* fusion with mutant *rbfL* start codon)	This study	N/A
prmp39 ($tRNA^{Arg(UCU)}$ locus with native promoter and downstream sequence in prabm1515)	This study	N/A
prmp40 ($tRNA^{Arg(UCU)}$ locus with native promoter and downstream sequence in prabm1516)	This study	N/A
prmp41 ($tRNA^{Arg(UCU)}$ locus with native promoter and downstream sequence in prabm1517)	This study	N/A
prmp42 (RKR to RKG quickchange in prabm1515)	This study	N/A
prmp44 (RKR to RKG quickchange in prabm1516)	This study	N/A
prabm91 (RKR to RKG quickchange in prabm1517)	This study	N/A
prabm98 (RKR to RKE quickchange in prabm1515)	This study	N/A
prabm99 (RKR to RKE quickchange in prabm1516)	This study	N/A
prabm101 (<u>R</u>KR non-rare quickchange in prabm1515)	This study	N/A
prabm102 (<u>R</u>KR non-rare quickchange in prabm1516)	This study	N/A
prmp35 (RKR to AKR quickchange in prabm1515)	This study	N/A
prmp36 (RKR to RAR quickchange in prabm1515)	This study	N/A
prmp37 (RKR to RKA quickchange in prabm1515)	This study	N/A
Software and algorithms		
Cryosparc v4.7	Punjani et al., (3)	https://cryosparc.com/
UCSF Chimera v1.17.2	Pettersen et al., (4)	https://www.cgl.ucsf.edu/chimera/
Pymol	Schrödinger (5)	https://www.pymol.org/
AlphaFold 3	Abramson et al., (6)	https://alphafoldserver.com/

Other		
Quantifoil R 2/2 300-mesh copper grids	Electron Microscopy Sciences	SKU: Q3100CR2

Supplementary References:

1. Charpentier, E. et al. Novel cassette-based shuttle vector system for gram-positive bacteria. Appl. Environ. Microbiol. 70, 6076–6085 (2004).
2. Helle, L. et al. Vectors for improved Tet repressor-dependent gradual gene induction or silencing in Staphylococcus aureus. Microbiology 157, 3314–3323 (2011).
3. Punjani, A., Rubinstein, J. L., Fleet, D. J. & Brubaker, M. A. cryoSPARC: algorithms for rapid unsupervised cryo-EM structure determination. Nat. Methods 14, 290–296 (2017).
4. Pettersen, E. F. et al. UCSFChimera—A visualization system for exploratory research and analysis. J. Comput. Chem. 25,1605–1612 (2004).
5. Schrödinger, L. PyMOL http://www.pymol.org/pymol (2020).
6. Abramson, J. et al. Accurate structure prediction of biomolecular interactions with AlphaFold 3. Nature 630, 493–500 (2024).

Statistics and Reproducibility

Toeprint experiments in figures S1, S5 and S7 are representative and were performed three times. The raw uncropped image of each supplementary toeprinting figure is provided in the Source data file.
