# Supplementary material for: Extended Shine-Dalgarno motifs govern translation initiation in Staphylococcus aureus
Source: Nat Commun. 2026 Feb 12;17:2678. doi: 10.1038/s41467-026-69079-8 (PMC13009471; doi:10.1038/s41467-026-69079-8)
Supplement: Supplementary file 2 — Description of Additional Supplementary Files [file 41467_2026_69079_MOESM2_ESM.pdf]

Description of Additional Supplementary Files:

Supplementary Data 1: Summary of small ORFs identified by Ribo-Ret in *S. aureus* HG001. The genomic location, identifier, strand position, SD-motif presence, exact amino acid sequence, start codon identity, assigned category, predicted transmembrane helix (TMH) presence and the results from toeprint validation are indicated for each listed sORF. Furthermore, detailed information about the results from conservation analysis by blastN and tblastN are provided alongside individual notes by the authors for each sORF.
